# What's good for the heart is good for the brain? Evidence from a rural cohort on the longitudinal relationship between atherosclerotic cardiovascular disease risk and cognitive decline

**DOI:** 10.1016/j.tjpad.2026.100672

**Published:** 2026-09-15

**Authors:** Maileen G. Ulep

**Affiliations:** Memory and Cognitive Disorders, Lou Ruvo Center for Brain Health, Cleveland Clinic Nevada, 888 W. Bonneville Ave., Las Vegas, NV 89106, United States

**Keywords:** AHA PREVENT, Cardiovascular disease, Dementia, Life-course, Rural population

Could unlocking the complex relationship between cardiovascular and brain health be a key factor in reducing the risk of or preventing dementia? A recently published study by Krishnan and colleagues (2026) shows that in a U.S. cohort of 1,283 young adults with a mean age of 23, approximately 80% met the criteria for cardiovascular-kidney-metabolic (CKM) syndrome [1]. CKM is associated with an earlier onset of cardiovascular disease (CVD), and its prevalence is increasing. The development of the American Heart Association (AHA) Predicting Risk of CVD EVENTs (PREVENT) equations emerged from the need to incorporate predictors of total CVD and CKM risk, and one that can be applied to younger populations [2]. This is highly relevant to the field of Alzheimer's disease and related dementias (ADRD) because CVD is a well-known risk factor for cognitive decline. Several of the modifiable risk factors for dementia noted in the 2024 Lancet Commission report on dementia prevention overlap with those of CVD, including diabetes, dyslipidemia, hypertension, obesity, physical inactivity, and smoking [3].

Due to an interplay of various factors—including socioeconomic and healthcare infrastructure limitations—the burden of dementia and CVD is disproportionately higher in rural compared to urban communities in the U.S. Despite this, rural populations remain understudied. In this context, the study by Dhanasekara and colleagues offers a timely and clinically relevant meaningful evaluation of the longitudinal relationship between cardiovascular risk and cognitive trajectory in a rural cohort via Project FRONTIER (Facing Rural Obstacles to Healthcare Now Through Intervention, Education, and Research) [4].

While cardiovascular disease risk at baseline is a known predictor of cognitive deterioration, a better understanding of how increasing risk over time influences cognitive function is needed. Dhanasekara and colleagues explored whether a higher atherosclerotic cardiovascular disease (ASCVD) risk predicts accelerated cognitive decline using a Bayesian linear mixed-effects model. The AHA PREVENT equations were utilized to calculate the 10-year risk of ASCVD, which incorporates systolic blood pressure, cholesterol levels, renal function, diabetes, smoking status, age, and biological sex. The study's primary outcome was global cognitive function, as assessed by the Repeatable Battery for the Assessment of Neuropsychological Status (RBANS) total score. Secondary outcomes included the Clock Drawing Test (CLOX), Executive Interview 25 (EXIT25), Trail Making Test Parts A and B (TMT-A and TMT-B), and verbal fluency (FAS).

The study revealed that each 10-percentage point increase in a participant's 10-year ASCVD risk was associated with an additional point of annual decline on the RBANS total score [4]. Additionally, a faster rate of decline in specific cognitive domains—namely attention and delayed memory—was associated with increasing ASCVD risk, consistent with the typical profile of vascular-related cognitive dysfunction. Participants with significantly lower ASCVD risk demonstrated a small improvement in RBANS scores over time. As noted by the authors, this may not necessarily reflect actual cognitive improvement, but rather retest-related learning effects. Sex-specific patterns emerged that align with existing literature on the cognitive profile of healthy older adults: a male advantage in visuospatial processing and a female advantage in memory and verbal learning. In this study, women scored higher than men on global and verbal domain of the RBANS, as well as the EXIT25, but lower on the CLOX and visuospatial/constructional tasks [4].

While there are limitations—including the observational design restricting causal inferences and a relatively short median follow-up of 3 years with only two assessments per participant—the study has several noteworthy strengths. It is novel in its use of the AHA PREVENT equations to assess the longitudinal relationship between ASCVD risk and cognitive trajectory. More specifically, the study explored this association in a rural cohort, an underrepresented population in the literature despite the high burden of CVD and dementia in these communities. Because this was an observational study consisting of volunteers, it is likely that the sample population is healthier and more motivated than the general population as the authors noted. Given this potential healthy-volunteer bias, an association between higher 10-year ASCVD risk and faster cognitive decline still emerged suggesting that this finding is likely robust.

With CVD emerging earlier in life in the setting that life expectancy is increasing, studies that further elucidate the relationship between cardiovascular and brain health have significant clinical and future research implications. From a clinical standpoint, this study underscores the importance of early assessment and intervention regarding cardiovascular risk to mitigate incipient cognitive decline. Crucially, the AHA PREVENT equations may serve as a valuable clinical tool for identifying patients at highest risk for future cognitive impairment. CVD and dementia both have long incubation periods before clinical symptoms manifest; however, CVD can present clinically decades before ADRD [5]. This positions CVD and its risk factors as potential critical predictors of future cognitive impairment. The study by Dhanasekara and colleagues exemplifies this utility by using the AHA PREVENT equations to explore this dynamic in a population highly susceptible to both conditions. Their work provides a starting point and framework for future research. Longer-term studies with imaging and biomarker data, and include more than two assessments per participants, across diverse populations are needed to confirm the authors' findings and advance our insights into the longitudinal relationship between cardiovascular risk and cognitive outcomes.

Given that atherosclerosis has a long, asymptomatic phase of development that starts early in life, including childhood [3,5], additional research is needed to explore the potential mechanisms and identify critical stages where trajectories diverge toward either optimal cardiovascular and brain health or chronic disease. Identifying these critical periods may reveal windows of opportunity for multidomain intervention strategies to be initiated earlier in the life course, ultimately helping to prevent or delay disease in adulthood, older age, and subsequent generations.

## Declaration of generative AI and AI-assisted technologies in the manuscript preparation process

There was no use of generative AI or AI-assisted technologies in scientific writing or in figures, images and artwork.

## CRediT authorship contribution statement

**Maileen G. Ulep:** Conceptualization, Writing – original draft, Writing – review & editing.

## Declaration of competing interest

The authors declare that they have no known competing financial interests or personal relationships that could have appeared to influence the work reported in this paper.
